# Antiretroviral initiation of pregnant women and antenatal care booking practices in eThekwini District, KwaZulu-Natal, South Africa

**DOI:** 10.4102/phcfm.v10i1.1606

**Published:** 2018-05-24

**Authors:** Nomonde Nozulu, Bernard M. Gaede

**Affiliations:** 1 Department of Family Medicine, University of KwaZulu-Natal, South Africa

## Abstract

**Background:**

The introduction of antiretroviral therapy (ART) in South Africa began as part of the prevention of mother-to-child transmission programme. For significant reduction of vertical transmission, early antenatal care booking and ART initiation are necessary.

**Aim:**

This study aimed to evaluate ART initiation and booking practices of women attending antenatal care in eThekwini district during financial years (FY) 2010/2011 and 2013/2014.

**Methods:**

An observational study used a retrospective chart review at four eThekwini district community health centres (CHC). From these CHCs, records of women that initiated ART in FY10/11 and FY13/14 were reviewed and compared for ART initiation delays and booking practices.

**Results:**

A total of 2749 pregnant women who attended antenatal care (ANC) at the study sites were found eligible for ART; of these, 49% (*n* = 1334) attended ANC in FY10/11 while 51% (*n* = 1414) attended in FY13/14. In FY10/11, 46% (*n* = 610) and 60 % (*n* = 855) of the women were initiated on ART during pregnancy. The mean gestational age at booking for FY10/11 was 20.88 (standard deviation [s.d.] = 5.6) and 18.40 (s.d. = 6.2) in FY13/14. The mean gestational age at ART initiation for women who initiated ART in FY10/11 was 26.30 (s.d. = 6.02) and in FY13/14 it was 19.06 (s.d. = 6.86).

**Conclusion:**

In FY13/14 ART initiations occurred within 4 days after booking. ANC booking before 20 weeks was found to have improved between the two years from 39% to 58%; however, on average, in both years women booked during the second trimester.

## Introduction

In sub-Saharan Africa, women of reproductive age are reported to be the most affected by the human immunodeficiency virus (HIV), and in South Africa the antenatal care (ANC) HIV prevalence for 2013 was reported to be 29.7% nationally and 40.1% for eThekwini District in the KwaZulu-Natal (KZN) province.^1^ Since the inception of the prevention of mother-to-child transmission (PMTCT) of HIV programme in the country, the South African Department of Health (DOH) has continually introduced reforms to ensure that pregnant women living with HIV receive antiretroviral therapy (ART) both for prophylaxis and optimisation of their own health at the right time with minimal delays.

Early ART initiation for eligible pregnant women is favoured as being more effective in improving immunological and viral responses compared to post-partum initiation when measured 6 months after initiation.^2^ According to Melekhin et al., possible reasons for the positive responses to ART in pregnancy may be linked to the close monitoring that pregnant women undergo during ANC.^2^

In an observational cohort study, Fitzgerald et al. established that women presented for their first ANC visit at a median gestational age of 28 weeks, with 25% of women presenting after 31 weeks. According to this PMTCT study, late ANC booking resulted in the women missing the necessary ART exposure for effective reduction of vertical transmission.^3^

Early ART initiation in pregnancy is associated with less vertical transmission. Black et al. found that more than 7 weeks of ART exposure before delivery resulted in an HIV transmission rate of 0.3%.^4^ In a retrospective cohort study (data from 2003 to 2010) that was conducted in Gugulethu, Cape Town, South Africa, by Myer et al., women who presented early for ANC were likely to be on ART by the time of delivery and thus had reduced vertical transmission chances.^5^ Eligible women who did not initiate ART during pregnancy were found to have booked for ANC at a median gestational age of 31 weeks, compared to 27 weeks for those that started treatment.^5^

Early initiation is linked to early ANC booking; thus the 2015 *Guidelines for Maternity Care in South Africa* recommend that women start ANC as early as possible, preferably during the first trimester (≤ 12 weeks gestation).^6^ The recommended booking gestational age was also pronounced in the *Basic Antenatal Care Guidelines* of 2007 as an appropriate time for a full obstetric assessment and early referral for management where risk factors are identified.^7^ HIV diagnosis is one of the assessments that pregnant women undergo during the first ANC visit and, according to the South African guidelines on maternal care, all HIV positive women should be started on ART as soon as an HIV positive status is determined.^6,8,9^

The 2014/2015 District Health Barometer reported the national ANC booking before 20 weeks to be 53%; while this figure fell below the set target of 65% it showed great improvement compared to previous years.^10^ For KZN the proportion of women who booked before 20 weeks was reported to be 57%, with eThekwini reported to be at 54%.^10^

The PMTCT programme, which was first introduced in 2002, depended on doctors for ART initiation and management of patients. Nurses working in ANC were tasked with identifying eligible clients and either referring them to the doctor in a secondary institution or having them wait for a visiting doctor. The 2010 ART guidelines called for the implementation of the nurse-initiated management of ART (NIMART) programme; this was aimed at minimising the dependency on doctors and reducing waiting time for patients.^11^ NIMART is a task-shifting programme that utilises professional nurses to initiate and manage patients on ART. The World Health Organisation (WHO) defines task shifting as the process of moving tasks to other categories of health workers.^12^ Task shifting has been reported to be cost-effective, offering good quality care to more patients compared to doctor-centred care.^13^ Nurses form 80% of healthcare professionals practising in South Africa and have for years been working independently within the primary health care setting.^14^ The introduction of NIMART expanded the health care skill set necessary for ART initiation, resulting in the decentralisation of these services for eligible pregnant women attending ANC and also removing the need to refer stable clients to doctors.^11^

For the country to have an effective PMTCT programme it was necessary to not only expand the human resource for ART initiations but also monitor ANC booking practices to ensure that clients booked as recommended so as to commence treatment when it’s most beneficial.

### The local context of the study

The implementation of PMTCT changed significantly with the introduction of the new NIMART policy. After the 2010 guidelines were released, the South African DOH collaborated with various non-governmental organisations to provide NIMART training in the country. Following the 5-day didactic NIMART training, nurses are required to undergo on-site mentorship under the guidance of a doctor or a nurse who is already certified to be competent in ART initiation. During NIMART mentorship the mentees are required to initiate a set number of patients in different categories, including paediatrics, pregnant women, adult men, women of childbearing age, as well as people living with HIV who have TB co-infection.^15^

**FIGURE 1:**
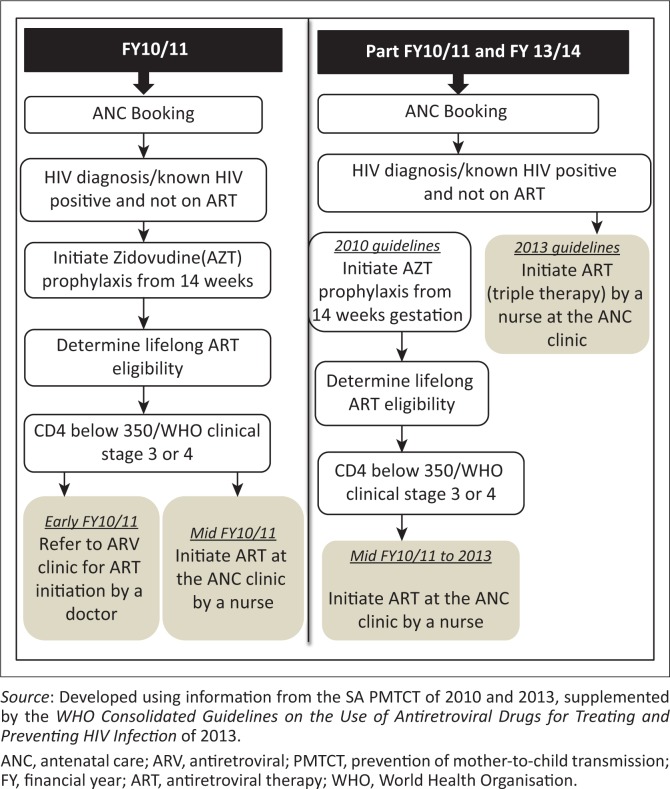
Differences in antiretroviral therapy management between FY10/11 and FY13/14 in eThekwini district community health centres.

The differences in ART initiation between the two time frames were determined by the country’s ART guidelines. The framework for the 2010 and 2013 guidelines is outlined in Figure 1.

The points of ART initiation are depicted with the grey boxes. During early FY10/11, after the announcement of the 2010 guidelines, pregnant women eligible for ART were referred to ART clinics for initiation by a doctor. When NIMART training was introduced in 2010, eligible ANC clients started receiving ART under the care of nurses.

### Aims and objectives

The aim of the study was to compare ART initiation of pregnant women attending ANC in community health centres (CHCs) of eThekwini district between financial years (FY) 10/11 and 13/14. Specific objectives included the following:

- to measure the waiting time from ANC booking to ART initiation between groups initiated before and during NIMART
- to compare the proportion of initiations in the antiretroviral (ARV) clinic (doctor-driven) against those taking place in the nurse-run ANC clinics during the two FY
- to identify ANC attendance practices between the two groups.

## Methods

### Study design

This observational study used retrospective chart review to compare the initiation of pregnant women on ART before and after implementation of NIMART. The retrospective study was conducted in four eThekwini district CHCs.

### Setting

The eThekwini health district is divided into three sub-districts, namely south, north and west. The district has eight CHCs and five of these are in the north, two in the west and one in the south. CHCs have been providing ART to eligible pregnant women since the inception of the ART programme. However, before NIMART, patients were referred to separate ART clinics (within the CHC) to be initiated on ART and have their care managed by the doctor. The implementation of NIMART allowed for ART initiation for pregnant women to be included in the ANC clinic.

### Sample size and sample strategy

The sample size was calculated using Intercooled Stata version 13. To demonstrate the difference in ART initiation practices between the two years; the statistical power was set at 80% with a two-sided alpha of 0.05. The selection of the sample size was informed by the District Health Information System data for the two study FY. According to the district data, 71% (*n* = 942) of eligible pregnant women initiated ART during FY10/11, while 77% (*n* = 2782) started in FY13/14.^16^ The difference between the initiation proportions presented by the district data was considered in calculating the sample size for the study. A sample of 850 records for each of the two study periods was proposed.

The study was conducted at four CHCs. The one CHC in the south was included and the others were selected randomly, with two CHCs from the north sub-district and one CHC from the west sub-district. Patients were selected if they had attended ANC at the facilities, were HIV positive and had initiated lifelong ART during their pregnancy. Patients were sampled consecutively at all the CHCs until the sample size was obtained.

### Data collection

A data collection tool covering study variables was designed by the researcher. The design of the data collection tool was informed by the DOH ART management data collection tools, mainly the HIV, AIDS, sexually transmitted infection (STI) and TB (HAST) clinical chart, and the variables from the chart were adapted for this study. The DOH tools on which the data collection tool is based already have established validity and reliability and are used to collect programme monitoring data. Study numbers were allocated to each participating CHC, ranging from CHC1 to CHC4 and within those facilities, each data collection tool was allocated a unique study number.

Study data were collected in September and October 2015. The review of patients’ records started with data from the ANC clinic where all obstetric and ART preparation information was collected. The Three Interlinked Electronic Register (commonly referred to as TIER.Net) gave information as to the date of initiation as well as blood results. The HAST clinical chart was consulted for further information on the patients’ history, baseline immunology, physical assessment, ART regimen and follow-up care.

### Data analysis

After data collection, each data collection tool was checked for completeness before capturing into Microsoft Excel. The capturing was followed by a process of data cleaning. Data codes were allocated to categorical variables for easy analysis once exported to the statistic program for analysis (e.g. FY10/11 = 1 while FY13/14 = 2, yes = 2, no = 1). The cleaned data was then transferred for analysis to the SPSS 23 statistic program.

The data were analysed by applying general descriptive and analytical statistics. The confidence interval for both groups under comparison was set at 95%. The mean and the standard deviation were used to measure the location and distribution of numerical variables between the two study timelines. The difference between the means of the two study timelines variables was tested using the independent sample *t*-test. The associations between categorical variables were tested using the chi-square test.

## Ethical considerations

Ethical approval to conduct this study was granted by the Biomedical Ethics Research Ethics Committee of the University of KwaZulu-Natal (reference number: BE0028/15). Additional permissions were obtained from the KwaZulu-Natal DOH research office (reference number: KZ_2015RP7_554), the eThekwini District DOH and from the managers of the selected CHCs.

The managers of the participating CHCs were assured that facility names would not be linked to the data and that each site’s confidentiality would be protected by identifying each with a number. The names CHC1 to CHC4 were used to code each site. Patients’ personal information was also protected, as their personal details were not captured by the data collection form.

## Results

Data on demographic, obstetric, immunological information as well as ART initiation characteristics of the study group were collected. During FY10/11, 610 women initiated ART and 855 in FY13/14. Table 1 displays a breakdown of the results according to the two FY.

**TABLE 1:** Characteristics of women initiated on antiretroviral therapy during pregnancy at four eThekwini district community health centres during FY10/11 (*n* = 610) and FY13/14 (*n* = 855).

Variable	FY10/11		FY13/14	
	Frequency	Percentage	Frequency	Percentage
**Maternal age at first ANC visit**				
< 18 years	14	2	19	2
18–24 years	146	24	231	27
25–29 years	220	36	281	33
30–34 years	154	25	204	24
≥ 35 years	76	12	120	14
**Gravid state**				
First pregnancy	159	26	185	22
Two pregnancies or more	451	74	670	78
**Gestational age at first ANC visit**				
Before 20 weeks	235	39	494	58
After 20 weeks	375	61	361	42
**CD4 cell count**	**0**	**0**
< 200	248	41	205	24
200–350	361	59	623	73
> 350	1	0	27	3
**Clinical staging**				
Stage 1	44	7	333	39
Stage 2	485	80	404	47
Stage 3	81	13	118	14
**Previous PMTCT**
Yes	104	17	268	31
No	506	83	587	69
**HIV status known at ANC booking**				
Yes	152	25	360	42
No	458	75	495	58
**Days from first ANC booking to ART initiation**				
< 7days	4	1	642	75
7–14 days	70	11	155	18
15–30 days	231	38	37	4
31–90 days	280	46	21	2
> 90 days	25	4	0	0
**Gestational age at ART initiation**				
First trimester	2	0	137	16
Second trimester	404	66	640	75
Third trimester	204	33	78	9
**Point of ART initiation**
ART clinic	610	100	29	3
ANC clinic	0	0	826	97

ANC, antenatal care; ART, antiretroviral therapy; PMTCT, prevention of mother-to-child transmission; FY, financial year; CD4, cluster of differentiation 4.

### Implementation of nurse-initiated management of antiretroviral therapy policy

The ART initiation of pregnant women took place at two service points, namely the ARV clinic where initiations are shared between the nurses and doctors or the ANC clinic where ARV initiations are managed by nurses. In FY10/11 100% (*n* = 610) of the pregnant women started ART at the ARV clinic. In comparison, in FY13/14, 97% (*n* = 826) were initiated in ANC, with 3% (*n* = 29) having been attended to at the ARV clinic. This measure reflects extensive implementation of the NIMART policy.

### Gestational age during first antenatal care visit

In FY10/11, 61% (*n* = 375) of initiated women booked after 20 weeks compared to 42% (*n* = 361) in FY13/14 who booked during the same period. These results show an improvement in late booking practices between the two years.

The mean gestational age for FY10/11 was 20.88 (standard deviation [s.d.] = 5.6), while that for FY13/14 it was found to be 18.40 (s.d. = 6.2). The mean difference for the two study timelines was 2.4. The difference between the two means was found to be statistically significant (*t* = 7.759, *p* ≤ 0.0001).

**FIGURE 2:**
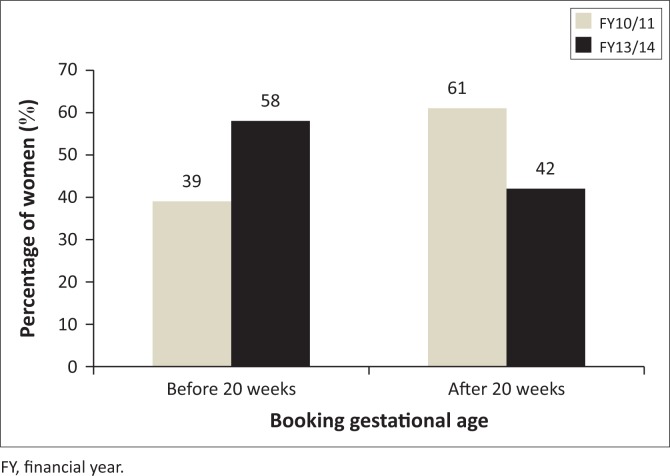
Antenatal care booking of women initiated on antiretroviral therapy at four eThekwini district community health centres in FY10/11 (*n* = 610) and FY13/14 (*n* = 855).

The distribution of the gestational age at first ANC visit between the two FY is shown in Figure 2 according to booking before and after 20 weeks.

### CD4 cell count and World Health Organisation clinical staging

The 1456 women who presented at the participating sites had a mean CD4 cell count of 239 cells. The mean for FY10/11 was 214.6 (s.d. = 78.05) while that for FY13/14 was 256.97 (s.d. = 82.65). The difference between the two means on an independent *t*-test was found to be –42.340. The *t*-test reflected a statistically significant difference between the two means (*t* = 9.98, *p* ≤ 0.0001).

**FIGURE 3:**
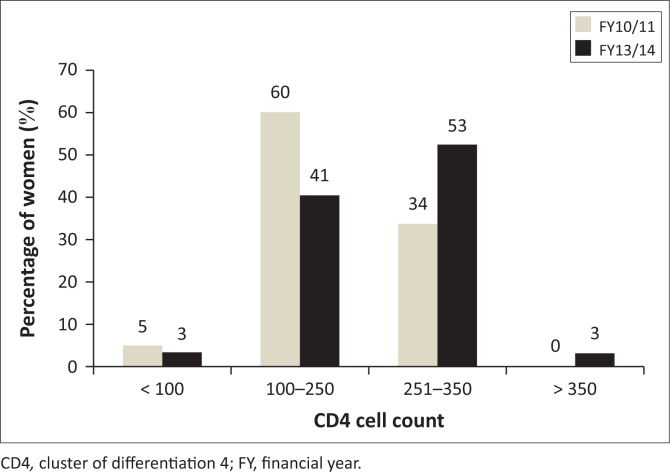
CD4 cell count distribution of women initiated on antiretroviral therapy in four eThekwini district community health centres during FY10/11 (*n* = 610) and FY13/14 (*n* = 855).

The CD4 cell count of women who initiated ART at participating CHCs for both FYs is displayed in Figure 3, ranging from less than 100 to more than 350.

During FY10/11, 60% (*n* = 369) of women were initiated with a CD4 cell count of 100–250 while 34% (*n* = 207) were found to have values between 251 and 350 cells. Fewer women were initiated with a CD4 below 5% (*n* = 33), while no women in FY10/11 had CD4 values above 350.

In FY13/14, 53% (*n* = 451) of women started ART with CD4 cell count values of 251–350, while 41% (*n* = 348) were situated in the 100–250 range. A lesser proportion of women, 3% (*n* = 29), had CD4 cell count results less than 100 and the same proportion was observed for the more than 350 category.

**FIGURE 4:**
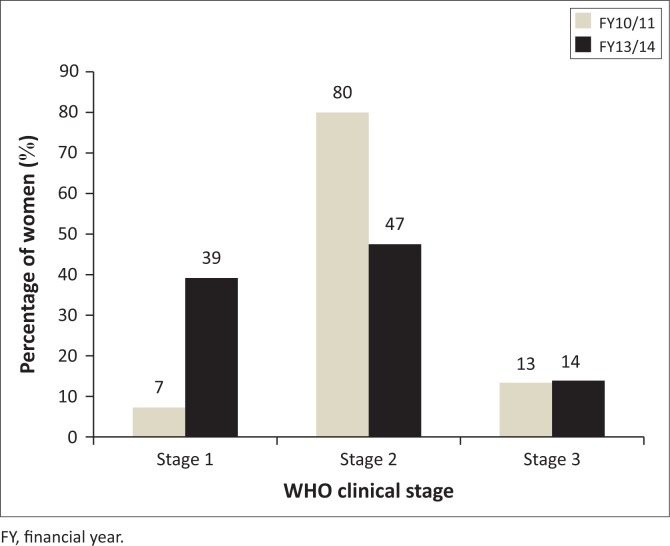
Distribution of World Health Organisation (WHO) clinical stage of women initiated on antiretroviral therapy in four eThekwini district community health centres in FY10/11 (*n* = 610) and FY13/14 (*n* = 855).

The results for WHO staging revealed a distribution between stages 1–3, and none of the clients were initiated during stage 4. The distribution of the WHO staging results from stages 1 to 3 is reflected in Figure 4.

During FY10/11, 80% (*n* = 485) of pregnant women were started on ART at WHO clinical stage 2, while 7% (*n* = 44) and 13% (*n* = 81) were initiated while at stages 1 and 3, respectively.

In FY13/14, 47% (*n* = 404) of women were initiated on ART while at WHO clinical stage 2 and 39% (*n* = 333) were at stage 1 when treatment was started. A proportion of 14% (*n* = 118) of women commenced ART while at WHO stage 3.

### Days from first antenatal care booking to antiretroviral therapy initiation

The ART initiation practices were measured in three variables, namely days from first ANC booking to ART initiation, gestational age at ART initiation and point of ART initiation. In FY10/11, the larger proportion of women started ART between 31 and 90 days after booking (46%; *n* = 280), while in FY13/14, 75% (*n* = 642) of women were initiated within 7 days. In both FYs women started ART during the second trimester, 66% (*n* = 404) in FY10/11 and 75% (*n* = 640) in FY13/14. Of those initiated during the third trimester, 33% (*n* = 204) were seen in FY10/11 compared to 9% (*n* = 78) in FY13/14.

The delay in the number of days from first ANC visit to the date of ART initiation for FY10/11 was 37.95 (s.d. = 28.71) while that for FY13/14 was 4.12 (s.d. = 9.64). The difference between the two means calculated with an independent *t*-test was 33.830; the test also revealed a statistically significant difference between the two means (*t* = 32.08, *p* ≤ 0.0001).

**FIGURE 5:**
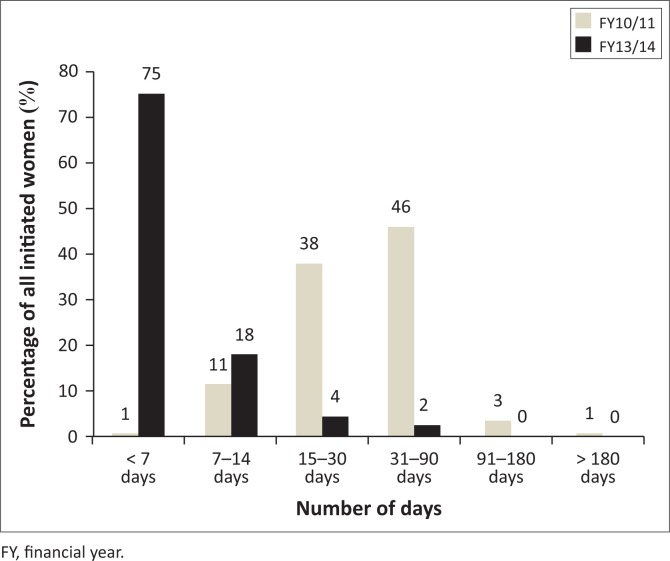
Distribution of days from first antenatal care visit to antiretroviral therapy initiation of women attending antenatal care in four eThekwini district community health centres during FY10/11 (*n* = 610) and FY13/14 (*n* = 855).

The number of days that the patients waited before initiating ART was divided into six categories of days ranging from less than 7 to more than 180 days. The results of this breakdown between the two FYs are displayed in Figure 5.

### Gestational age at antiretroviral therapy initiation

The mean gestational age at ART initiation for women who initiated ART in FY10/11 was 26.30 (s.d. = 6.02), while on average in FY13/14 the women started ART at 19.06 (s.d. = 6.86) weeks gestational age. An independent *t*-test showed a mean difference of 7.2 as well as a statistically significant difference between the two means (*t* = 20.94, *p* ≤ 0.0001).

**FIGURE 6:**
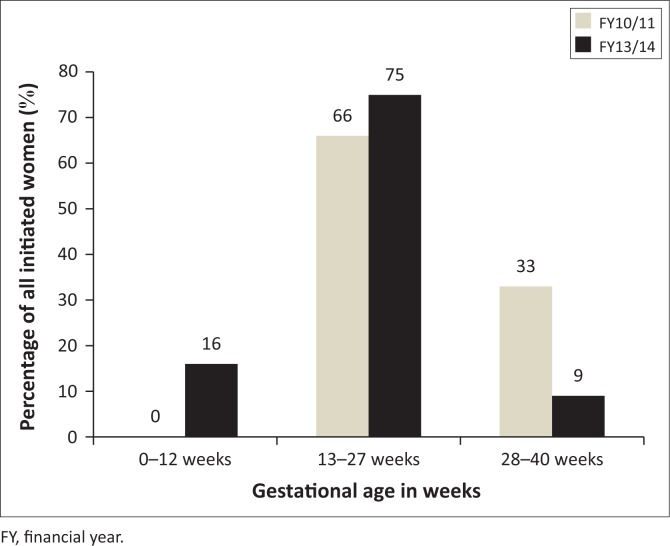
Distribution of gestational age at antiretroviral therapy initiation according to trimester of women initiated on antiretroviral therapy during pregnancy at four eThekwini district community health centres in FY10/11 (*n* = 610) and FY13/14 (*n* = 855).

The distribution of gestational age at ART initiation for both FY is reflected in Figure 6 according to trimester.

In both FY the gestational age at ART initiation clustered between 13 and 27 weeks gestation. Within the second trimester, the mean gestational age was 20 weeks for FY10/11, while those in FY13/14 initiated ART at a mean of 19 weeks.

### Previous prevention of mother-to-child transmission and antenatal care booking

A total of 372 women who started ART at the study CHCs were recorded to have had some exposure to PMTCT in the past. Of these women, 104 initiated ART in FY10/11 while 268 were seen in FY 13/14.

**FIGURE 7:**
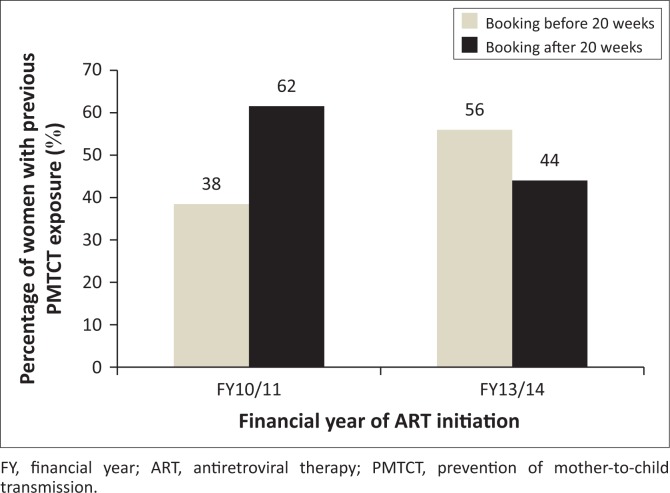
Previous prevention of mother-to-child transmission exposure and antenatal care booking practices of women initiated on antiretroviral therapy in eThekwini district in FY10/11 (*n* = 104) and FY3/14 (*n* = 268).

The data on the ANC booking practices of this group are shown in Figure 7.

Of the 104 women with a history of PMTCT exposure 62% (*n* = 64) booked for ANC after 20 weeks gestation while the remaining 38% (*n* = 40) presented before 20 weeks. In FY13/14, 56% (*n* = 150) of the 268 women with previous PMTCT exposure started ANC before 20 weeks gestation while a lesser 44% (*n* = 118) booked after 20 weeks.

The association between previous exposure to PMTCT and booking before 20 weeks was tested using a chi-square test. The hypothesis was that women with previous PMTCT exposure would book for ANC before 20 weeks. For FY10/11 the observed relationship between the variables was non-significant (*X*^2^ = 0.210, *p* = 0.988). The relationship was also found to be non-significant in the FY13/14 data (*X*^2^ = 0.523, *p* = 0.470). Therefore, for both FY under observation, previous exposure to PMTCT was not associated with early ANC booking.

### Known human immunodeficiency virus status at antenatal care booking

**FIGURE 8:**
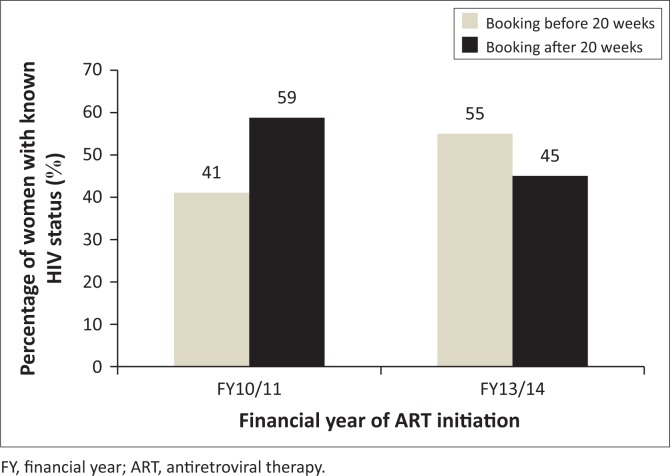
Human immunodeficiency virus (HIV) status knowledge at first antenatal care visit and booking practices of women initiated on antiretroviral therapy in eThekwini district during FY10/11 (*n* = 152) and FY13/14 (*n* = 360).

Approximately 512 women who initiated ART at the four CHCs already knew they were living with HIV when they booked for ANC. The distribution of the data for booking before 20 weeks and after 20 weeks for those who knew their status is reflected in Figure 8.

Of the 512 women, 30% (*n* = 152) were seen in FY10/11 while the remaining 70% (*n* = 360) attended ANC in FY13/14. In FY10/11, 59% (*n* = 89) of women with known HIV status at booking started ANC after 20 weeks while 41% (*n* = 63) presented before 20 weeks. Data from FY13/14 showed 55% (*n* = 199) of women with known status to have booked before 20 weeks.

The association between known HIV status at booking and ANC booking before 20 weeks was tested using the chi-square statistic. The hypothesis was that knowing one’s HIV status before ANC booking would result in women booking before 20 weeks. For both FYs the relationship was found to be statistically non-significant. For FY10/11 the chi-square test for the observed relationship was *X*^2^ = 0.730, *p* = 0.393. The chi-square test for FY13/14 was *X*^2^ = 1.59, *p* = 0.207.

## Discussion

### Gestational age at antenatal care booking

In South Africa, booking before 20 weeks (preferably during the first trimester) is recommended to allow for early maternal health assessments and interventions, including ART initiation for those found eligible.^6,7^ In the Strategic Plan for Maternal, Newborn, Child and Women’s Health and Nutrition in South Africa 2012–2016, the target for women booking for ANC before 20 weeks for 2013 was 55% and this was set from a baseline of 34%.^8^

During the study period of FY10/11, the women booked for ANC at a range of 8–36 weeks gestation compared to 6–39 found in FY13/14. When one looks at this indicator in terms of booking before or after 20 weeks, the results show a 29% improvement in booking before 20 weeks between the two years. In FY10/11 39% (*n* = 235) of women were reported to have booked before 20 weeks while 58% (*n* = 494) in FY13/14 booked within this time frame. Although the women booked within the recommended period (before 20 weeks), there appears to be a preference for the second trimester booking and this resulted in the majority of the women starting ART during this gestational period.

Previous studies have reported similar results on gestational age booking. For instance, in a study on ART initiation, Stinson et al. reported a booking gestational age median of 26 weeks with a range of 21–31 weeks.^17^ Similar results were found by Myer et al. in another Cape Town study, where a median booking gestation age of 28 weeks was reported.^5^ Though these studies were conducted between 2005 and 2008, it’s interesting to note the continued practice of booking after the first trimester years later as shown in the current study. Compared to the other two studies referred to above, in a 2010 study, Van Schalkwyk et al. reported a lower average booking gestational age of 17 weeks, comparable to booking gestational ages of 18 and 20 weeks presented in this study.^18^

According to the District Health Barometer 2014/2015, the South African national average for booking before 20 weeks was 53%, below the target of 65%.^10^ The results from this study showed an improvement in booking practices before 20 weeks between the two FYs, with 19% (*n* = 259) more clients in FY13/14 found to have booked during this period compared to FY10/11. In addition, the 58% (*n* = 494) who booked before 20 weeks in FY13/14 presented a 5% improvement when compared to the national average of 53%. The performance in this indicator for FY13/14 was also slightly above the 2013 target of 55% set in the Strategic Plan for Maternal, Newborn, Child and Women’s Health and Nutrition in South Africa 2012–2016.

### Days from first antenatal care booking to antiretroviral therapy initiation

The delay in ART initiation was measured in days from the first ANC visit (booking) to the date of ART initiation. The number of days waited ranged from less than 7 days to more than 180 days. On average, women who initiated ART in FY10/11 waited approximately 38 days before receiving ART, compared to 4 days for the FY13/14 group, which is a difference of 34 days. Though these results show some improvements between the two study periods, they fell short of the guideline recommendations, which stipulate ART initiation at the point of diagnosis.^9^

In a comparative study by Van Schalkwyk et al., the duration between booking and ART initiation was measured in weeks and the median weeks for 2010 was 5.1, which translates to 35 days of waiting.^18^ This waiting time is similar to the 38-day delay presented in this study for FY10/11. In their observational cohort study conducted in August 2012 to February 2013 on PMTCT-related delays, Schnippel et al. reported that about 21% of patients initiated ART within 60 days while only 3% were initiated within 30 days.^19^ When compared to previous studies, this study revealed far fewer waiting days from booking to ART initiation.

### Gestational age at antiretroviral therapy initiation

In this study, the mean gestational age for ART initiation in FY10/11 was 26 weeks, while lower at 19 weeks for FY13/14. The study results showed a trend of relatively late ANC booking, resulting in women starting ART after the first trimester. The 26 weeks gestation for ART initiations is similar to other studies, with 25 weeks reported by Van Schalkwyk et al. and Schnippel et al. reporting 27 weeks.^18,19^ The gestational ages for ART initiations in the second trimester mentioned in these studies and FY10/11 of this study showed an improvement from the median gestational age of 32 weeks reported by Stinson et al.^17^

Noted as an improvement in this study is the gestational age at ART initiation between the two FYs, from a mean of 26 weeks in FY10/11 to 19 weeks in FY13/14; although it is in the second trimester it is still in the recommended time frame of before 20 weeks. It must be pointed out that, even though the women started therapy after the first trimester, they however managed to receive more than 8 weeks of ART by the time of delivery (as recommended by Black et al.) and thus had reduced chances of vertical transmission.^4^

### Antenatal care booking practices in relation to history of human immunodeficiency virus

Booking at advanced gestational age for women living with HIV means shorter ART exposure and thus increased chances for vertical transmission.^3,4^ In this study ANC booking was explored in relation to women who were reported to have been known HIV positive at first ANC visit as well as those with previous history of PMTCT.

### Previous prevention of mother-to-child transmission exposure

When looking at previous PMTCT exposure one might hypothesise that experience would have created more awareness, resulting in presenting earlier for ANC during subsequent pregnancies to received adequate management.

Of the 1465 women who initiated ART in FY10/11 and FY13/14, 25% (*n* = 372) were found to have been part of the PMTCT programme in previous pregnancies. In FY10/11, 62% (*n* = 64) of women in this group booked after 20 weeks, compared to 44% (*n* = 118) of women seen in FY13/14 who booked during this time frame. These results also show an 18% improvement in booking before 20 weeks in this group, from 38% (*n* = 40) in FY10/11 to 56% (*n* = 150) in FY13/14. As with known HIV status, these results are comparable to the rest of the study population.

### Known human immunodeficiency virus status

HIV testing and counselling is available at every point of care in South African health facilities through the Provider Initiated Counselling and Testing programme.^20^ According to this programme, all patients seeking health care must be given an opportunity to test for HIV. This is aimed at increasing the number of people who know their HIV status, and this would further facilitate early interventions. This also means that women starting ANC would have gone through HIV testing at some point if they had attended a health facility in the past.

For this study known HIV status was defined as being aware of one’s HIV positive status at the time of ANC booking. The results revealed that 512 women (for both timelines) were aware of their HIV positive status at the time of booking, with 49% (*n* = 250) of these having presented for ANC after 20 weeks gestation while the remaining 51% (*n* = 262) started ANC before 20 weeks.

When looking at the two FYs separately, 55% (*n* = 199) in FY13/14 booked before 20 weeks compared to 41% in FY10/11. These results show a 14% improvement in ANC booking of women with known HIV status. Also, the booking results for this group are comparable to the 58% achieved by the rest of the study population. These improvements are encouraging and if sustained and enhanced further could result in positive maternal health outcomes.

## Study limitations

### Selection bias

Study sites were selected according to geographical position, thus other factors such as the patient headcount per site, site-related staff shortages and the socio-economic conditions of the communities served were not considered. These differences between the participating sites can be considered to infer a selection bias and make the generalisability of the results to all district CHCs not possible.

### Information bias

Incomplete data and missing records posed information bias. This was first noted during the pilot study, where some study variables had to be refined to accommodate available data. This was observed mostly with laboratory results, which were inconsistently recorded. The challenge of poor recoding was consistent across all CHCs, with some sites being poorer than others.

## Conclusion

The introduction of NIMART in the PMTCT programme was meant to result in improved quality of care for pregnant women living with HIV. The study revealed a shift from ARV clinic initiations to the ANC; this is not only convenient for the women but also has potential positive benefits for the mothers and their unborn babies. In this study more women in FY13/14 were recorded to have accessed ART in the ANC clinic, compared to FY10/11, where all women were found to have been initiated at the centralised ARV clinic.

The early timing of ART initiation in pregnancy is associated with reduced vertical transmission. The benefits of early ART initiation are more often observed when treatment is started at an earlier gestational age. An improvement was noted in terms of ART initiation gestational age, with those in FY13/14 starting treatment earlier, at an average of 19 weeks compared to 26 weeks in FY10/11. The study showed an improvement in the timing of ART initiation after booking between the two timelines, with those initiated in FY13/14 starting ART at an average of 4 days after ANC booking compared to 38 days in FY10/11. This is an encouraging result and infers improvements in the management of pregnant women living with HIV.

### Possible or future studies

Qualitative studies on ANC booking practices would assist in gaining more knowledge on factors associated with late booking. Studies can be conducted to understand the knowledge, beliefs and attitudes on the subject from both the general population and health care workers. This knowledge would assist in creating targeted interventions.

Studies on nurse experiences and challenges with task shifting are also recommended; both qualitative and quantitative studies would be useful in this regard. In addition, such studies should be extended to include doctors to gauge their knowledge, attitudes and beliefs about task shifting.
